# Awareness of medical law among health care practitioners in Saudi Arabia

**DOI:** 10.15537/smj.2022.43.8.20210920

**Published:** 2022-08

**Authors:** Abdulelah S. Bin Shihah, Abdulrahman H. Alrashed, Khaled A. Al-Abduljabbar, Hassan A. Alamri, Mohammed Shafiq, Mayada N. Ahmed, Abdullah H. Alkhenizan

**Affiliations:** *From the Department of Family Medicine & Polyclinics (Bin Shihah, Alrashed, Al-Abduljabbar, Shafiq, Ahmed, Alkhenizan), King Faisal Specialist Hospital and Research Centre, Riyadh; and from the Department of Internal Medicine (Alamri), King Abdallah Hospital, Bisha, Kingdom of Saudi Arabia*.

**Keywords:** medical law, healthcare, Saudi Arabia

## Abstract

**Objectives:**

To determine the level of awareness of medical law among healthcare practitioners and to identify factors that influence that level of awareness in Saudi Arabia.

**Methods:**

This cross-sectional study was carried out in Riyadh, Saudi Arabia in 2020-2021 via a survey including 750 healthcare practitioners, from different specialties including all regions in Saudi Arabia. Participants included consultants, senior registrars, interns, and residents.

**Results:**

Majority of enrolled healthcare practitioners had poor awareness of medical law (approximately 97%). Only 1.5% had adequate awareness of medical law, and only 1.5% had moderate awareness. Factors associated with increased medical law awareness were the age group between 25 and 34, being from the central region, and having a clinical practice for less than 10 years *p*-value of <0.05.

**Conclusion:**

Awareness of medical law among healthcare practitioners is limited in Saudi Arabia. Professional medico-legal education should be part of required competencies for undergraduate and postgraduate levels of medical education. Health care practitioners must be educated with laws and regulations of practicing health professions in the country.

**H**ealthcare sector development has been prioritized by Saudi Arabia’s Vision 2030, which focuses on policy, health, social, and economic development.^1,2^ Healthcare is a fundamental right in Saudi Arabia. Saudi Arabia has established a deep-rooted health system, which provides free health services for citizens by adopting welfare policy and universal health care coverage.^3,4^ Adherence to the standards of medical practice and thorough awareness of medical law are pivotal to reduce medical errors, clinical negligence and litigations against medical practitioners.^5^ Medical practice litigation has increased in recent years due to the increasing awareness of medical and legal matters.^6^

Medical errors evaluated and announced by the Ministry of Health (MOH) in Saudi Arabia seems to have a positive impact on medical practitioners’ awareness of medical law.^5,7^ There has been an increase in medical litigation alongside the development of health care services in Saudi Arabia.^5^ Public demands and awareness have contributed to the growing need to raise medical care practitioners’ awareness of medical law. Competencies covering in-depth understanding of health related medicolegal regulations are equally as essential to medical practice as basic sciences or clinical skills competencies. These competencies can potentially reduce medical errors and improve the delivery of safe health care services.^8^

There are local or regional studies have addressed the medical practitioners’ awareness of medical law; there has been substandard reported awareness of medical law in the region.^9-11^ Inadequate awareness of medical law can put medical practitioners at risk of violating the patients’ rights committing medical errors and clinical negligence.^12^

## Methods

This study was carried out in Riyadh, Saudi Arabia in 2020-2021, including consultants, senior registrars, interns, and residents. The calculated sample size equation suggested by Schaeffer and Mendenhall^13^ was used to estimate the proportion of the targeted population, which was 384 participants using a 95% confidence interval (CI) and a 5% margin of error. Inclusion criteria were all health care practitioners in all regions of Saudi Arabia, participants included consultants, senior registrars, interns, and residents, and exclusion criteria were healthcare practitioners who are not currently practicing in Saudi Arabia, or younger than 18 years of age. Data collection via face validity questionnaire that consisted 3 sections was developed by a group of experts and. The first section contained demographic details on the subjects. Second, included 5 questions on their knowledge and previous education in medical law and ethics. Lastly, it included 22 questions that addressed medical law awareness. The survey was in English language, delivered and disseminated to the participants online, however, some of the participants interviewed face-to-face.

Those who responded to 11 out of 22 questions correctly were categorized as having adequate awareness, those who answered between 7 and 10 questions correctly were tagged as moderately aware, and those who answered below 7 questions correctly were considered to have poor medical law awareness. This categorization was classified based on the Code of Ethics for Healthcare Practioners by The Saudi Commission of Health Specialties (Department of Medical Education and Postgraduate Studies) after many discussions with a group of experts in the medical law field.

The search method used to find previous related research based on the purpose of this study used a shared model of literature search model. The term medical law was used to identify relevant literature in the review and to determine the factors affecting the awareness of health professions in Saudi Arabia.

### Statistical analysis

Data were analyzed using the Statistical Package for the Social Sciences, version 26 (IBMCorp, Armonk, NY, USA). Fisher-Freeman-Halton test was used to test the significant relationships. A *p*-value of <0.05 was considered significant to test a hypothesis based on a difference between sample means.

## Results

A total of 750 health care practitioners enrolled in this cross-sectional study which was carried out in one of the main training center in Saudi Arabia. The majority of participants (422) were between 25 and 34 (56.3%) years old, 513 (68.4%) were male, 663 (88.4%) were Saudi, and 358 (47.7%) were medical doctors by specialty. They were distributed across various institutions, but most of them, specifically 295 (39.3%), represented Ministry of Health hospitals, and 420 (56%) of them practiced in the central region.

In this study, 57 (7.6%) interns, 157 (20.9%) junior residents, 160 (21.3%) senior residents/registrar, and 66 (8.8%) consultants participated. More details on general characteristics of the health profession are available in Table 1. Moreover, the awareness of medical law among healthcare practitioners in Saudi Arabia was evaluated in Table 2, which included factors affecting their awareness level.

**Table 1:** - *General characteristics of the health profession. Awareness of medical law among healthcare practitioners in Saudi Arabia, 2021*.

Variables	n	%
***Age***		
18-24	123	(16.4)
25-34	422	(56.3)
35-44	128	(17.1)
45-54	54	(7.2)
55-64	22	(2.9)
65+	1	(0.1)
***Gender***		
Female	237	(31.6)
Male	513	(68.4)
***Nationality***		
Non-Saudi	87	(11.6)
***Specialty***		
Saudi	663	(88.4)
Dentist	67	(8.9)
Medical doctor	358	(47.7)
Nurse	96	(12.8)
Pharmacist	40	(5.3)
Student	85	(11.3)
Other	104	(13.9)
***Regions***		
Central region	420	(56.0)
Eastern region	50	(6.7)
Northern region	25	(3.3)
Southern region	167	(22.3)
Western region	88	(11.7)
***Institution***		
King Faisal Specialist Hospital	71	(9.5)
Military hospitals	111	(14.8)
Ministry of health hospitals	295	(39.3)
Other	113	(15.1)
Private hospitals	40	(5.3)
Security forces hospitals	16	(2.1)
University hospitals	104	(13.9)
***Year in clinical practice***		
Less than 10 years	616	(82.1)
More than 10 years	134	(17.9)
***Professional level***		
Not a physician	305	(40.7)
Intern	57	(7.6)
Consultant	66	(8.8)
Junior resident	157	(20.9)
Senior resident/registrar	160	(21.4)

Only 11 (1.5%) out of 750 had an adequate level of awareness. Additionally, another 11 (1.5%) out of 750 had moderate awareness of the medical law. The majority of the health care practitioner, specifically 728 out of 750, had poor awareness of medical law. There was a significant association between the level of knowledge and general factors such as age, region, years of clinical practice, and professional level of the practitioner (*p*<0.05). Adequate and moderate awareness of medical law in Saudi Arabia was more prominent among the 25- to 44-year-old group, those from the central region, had practiced for fewer than 10 years, and among the professional level of assistant consultants or senior registrars (*p*<0.05).

Only 24.8% respondents had read the Saudi Law of Practicing Healthcare Professions 1441h. During their educational journey, only 248 out of 750 were exposed to concepts related to medical law and regulations in Saudi Arabia. The Code Ethics for Healthcare Practitioners from the Saudi Commission for Health Specialties was read by 406 out of 750 respondents (54.1%). Among the participants 71 (9.5%) have been threatened to be sued, and 18 (2.4%) have been sued already in Saudi Arabia.

**Table 2:** - *Factors affecting awareness of medical law among healthcare practitioners in Saudi Arabia, 2021 (N=750)*.

Characteristics	Awareness			Total	*P*-value
	Adequate	Moderate	Poor	n (%)	
***Age***					
18-24	0	2	121	123 (16.4)	0.009
25-34	3	4	415	422 (56.3)	
35-44	3	3	122	128 (17.1)	
45-54	4	1	49	54 (7.2)	
55-64	1	1	20	22 (2.9)	
65+	0	0	1	1 (0.1)	
***Gender***					
Female	1	4	232	237 (31.6)	0.302
Male	10	7	496	513 (68.4)	
***Nationality***					
Non-Saudi	1	1	85	87 (11.6)	1.0
Saudi	10	10	643	663 (88.4)	
***Specialty***					
Dentist	2	2	63	67 (8.9)	0.330
Medical doctor	6	6	346	358 (47.7)	
Nurse	1	2	93	96 (12.8)	
Other	0	1	103	104 (13.9)	
Pharmacist	2	0	38	40 (5.3)	
Student	0	0	85	85 (11.3)	
***Regions***					
Central region	3	6	411	420 (56)	0.028
Eastern region	0	0	50	50 (6.7)	
Northern region	3	0	22	25 (3.3)	
Southern region	4	2	161	167 (22.4)	
Western region	1	3	84	88 (11.7)	
***Institutions***					
King Faisal Specialist Hospital	0	1	70	71 (9.5)	0.704
Military hospitals	3	0	108	111 (14.8)	
Ministry of health hospitals	5	5	285	295 (39.3)	
Other	3	2	108	113 (15.1)	
Private hospitals	0	0	40	40 (5.3)	
Security forces hospitals	0	0	16	16 (2.1)	
University hospitals	0	3	101	104 (13.9)	
***Year in clinical practice***					
Less than 10 years	8	7	601	616 (82.1)	0.133
More than 10 years	3	4	127	134 (17.9)	
***Professional level of training***					
Not a physician	6	6	293	305 (40.7)	0.032
Intern	2	0	55	57 (7.6)	
Consultant	0	0	66	66 (8.8)	
Junior resident	2	4	151	157 (20.9)	
Senior resident/Registrar	1	1	158	160 (21.4)	

**Table 3:** - *Experience of healthcare practitioners in Saudi Arabia in relation to medical law*.

Questions	Response	n	%
Have you ever read “The Saudi Law of Practicing Healthcare Professions 1441H”?	Yes	186	(24.8)
Have you ever studied Saudi medical law during your educational journey?	Yes	248	(33.1)
Have you ever read the “Code Ethics for Healthcare Practitioners” from the Saudi Commission for Health Specialties?	Yes	406	(54.1)
Have you ever been threatened to be sued in Saudi Arabia?	Yes	71	(9.5)
Have you ever been sued in Saudi Arabia?	Yes	18	(2.4)

## Discussion

This study evaluated levels of awareness of medical law among healthcare practitioners in Saudi Arabia and identified factors that influence these levels. The included samples represented the different levels of healthcare practitioners’ genders, and nationalities; in addition, they were from various types of institutions.

A recent study from Saudi Arabia examined medical malpractice litigation in Saudi Arabia and compared it to that in the United Kingdom. It demonstrated an increase in rates of medical errors in both Saudi Arabia and the United Kingdom. It noted that the main sources of potential medical errors litigated were surgical specialties.^6^

In the literature, there were few studies addressing doctors’ awareness of medical law.^6^ In this study, most health care practitioners (97%) had poor awareness of medical law. Only 1.5% had adequate awareness of medical law, and only 1.5% had a moderate degree of awareness. The percentage found in this study is low, but expected as education for healthcare practitioners in the field of medical law is scarce and almost non-existing in the country. There are no local studies to compare. Therefore, compared to this study they were categorized as they have an adequate knowledge on medical ethics and medico-legal issues.

Few studies that have addressed the same issues have reported more than this study’s average regarding medical care practitioners’ awareness of medical law.^9,10^ Further explanations were explored in this area with sufficient explanations and discussions.^14^

A recent study from Australia assessed the current doctors’ understanding and awareness of the legal requisites, medical law, and patients’ consent to medical procedures. The findings suggest that only a third of the doctors were aware that a Court of Law defines the reasonable standard of care concerning obtaining informed consent. Less than half of them were aware of the High Court of Australia’s definition.^15^ These findings were comparable with a study which assessed the doctors’ knowledge and practice of legal and healthcare standards in practice settings.^15^ They have revealed complex matters concerning healthcare practitioners in relation to the legal context and consent and medical law-related issues.^16^

The poor awareness of medical law reported in this study was possible due to the lack of continuous professional training in medical law and limited specialist medical law availability. An insufficient awareness of medical law is likely to lead to poor service quality, and the practitioners will be at risk of being held liable, thus precipitating medical litigations.

According to the results of this study addressing legal regulations in place that are unclear and make interpretation difficult, this circumstance exposes medical professionals with poor perception to the possibility of legal liability. This situation puts healthcare practitioners with inadequate perception at the potential risk of legal responsibility. The factors that affected medical law awareness included 25 to 34 years old, from the central region, having had a clinical practice for less than 10 years, and an assistant consultant or registrar (*p*<0.05). These factors are needed to be considered in any future intervention. These findings are comparable with a British study which considered psychological medicine among Britain’s medical education.^17^

Professional legal standards should be taught in undergraduate courses, continuous professionals’ courses, and in post-graduate structured education programs. Awareness sessions is important in raise the awareness toward medical law.

### Study limitations

Due to current circumstances, namely, the COVID-19 pandemic, the course of professional training has rarely been organized. Therefore, lost the opportunity to attend awareness sessions before data collection.

In conclusion, awareness of medical law among healthcare practitioners was found to be below the optimum in most of the studied population in Saudi Arabia. Professional legal standards should be taught in undergraduate courses, continuous professionals’ courses, and in post-graduate structured education programs. Awareness sessions is important in raise the awareness toward medical law.
